# Identification and validation of a novel ferroptosis-related gene model for predicting the prognosis of gastric cancer patients

**DOI:** 10.1371/journal.pone.0254368

**Published:** 2021-07-12

**Authors:** Gang Liu, Jian-ying Ma, Gang Hu, Huan Jin

**Affiliations:** 1 Department of Breast Surgery, Thyroid Surgery, Huangshi Central Hospital of Edong Healthcare Group, Hubei Polytechnic University, Huangshi, Hubei, China; 2 Department of Pathology, Huangshi Central Hospital of Edong Healthcare Group, Hubei Polytechnic University, Huangshi, Hubei, China; Sapporo Ika Daigaku, JAPAN

## Abstract

**Background:**

Ferroptosis is a novel form of regulated cell death that plays a critical role in tumorigenesis. The purpose of this study was to establish a ferroptosis-associated gene (FRG) signature and assess its clinical outcome in gastric cancer (GC).

**Methods:**

Differentially expressed FRGs were identified using gene expression profiles from The Cancer Genome Atlas (TCGA) and Gene Expression Omnibus (GEO) database. Univariate and least absolute shrinkage and selection operator (LASSO) Cox regression analyses were performed to construct a prognostic signature. The model was validated using an independent GEO dataset, and a genomic-clinicopathologic nomogram integrating risk scores and clinicopathological features was established.

**Results:**

An 8-FRG signature was constructed to calculate the risk score and classify GC patients into two risk groups (high- and low-risk) according to the median value of the risk score. The signature showed a robust predictive capacity in the stratification analysis. A high-risk score was associated with advanced clinicopathological features and an unfavorable prognosis. The predictive accuracy of the signature was confirmed using an independent GSE84437 dataset. Patients in the two groups showed different enrichment of immune cells and immune-related pathways. Finally, we established a genomic-clinicopathologic nomogram (based on risk score, age, and tumor stage) to predict the overall survival (OS) of GC patients.

**Conclusions:**

The novel FRG signature may be a reliable tool for assisting clinicians in predicting the OS of GC patients and may facilitate personalized treatment.

## Introduction

Gastric cancer (GC) is a major malignant tumor with an incidence of 5.7% and a mortality rate of 8.2% based on global cancer statistics in 2018. Despite continuous improvements in surgery, advanced imaging techniques, and the development of new drug, an inefficient early diagnosis rate may result in a limited five-year survival in cases of GC [1,2]. The early stages of GC are usually asymptomatic, leading to a delayed diagnosis and missed opportunities for radical surgery. Therefore, there is an urgent need to discover more effective biomarkers for early diagnosis, therapeutic strategies, and prognostic assessment.

Ferroptosis is an iron-dependent non-apoptotic form of cell death because of the impaired repair of oxidized polyunsaturated fatty acids, redox-active iron, and lipid peroxides [3]. Ferroptosis has been reported to be a part of many pathophysiological processes, including the development of many types of cancers [4]. Various studies have revealed that ferroptosis can inhibit tumor growth and development, and ferroptosis induction inhibits the progression of chemotherapy-resistant tumors [5,6]. Multiple genes, such as P53 [7], RSL3 [8], FSP1 [9], and SLC7A11 [10], modulate sensitivity to or are markers of ferroptosis. However, the relationship between these ferroptosis-related genes (FRGs) and the prognosis of GC patients remains unclear.

In the present study, we collected RNA-seq data and the corresponding clinical data from public databases. We established a novel FRG signature for the prediction of OS in GC, which showed a favorable predictive capacity in the stratification analysis. A high risk score was associated with advanced clinicopathological features and an unfavorable prognosis. A genomic-clinicopathologic nomogram integrating risk scores and clinicopathological features was developed and validated. Therefore, the current investigation may further the exploration of prognostic FRGs and provide new insights into the possible mechanisms for GC development.

## Materials and methods

### Data obtained and analysis

The RNA sequencing data were obtained from The Cancer Genome Atlas STAD project (TCGA-STAD, https://cancergenome.nih.gov/), including 375 gastric adenocarcinoma samples and 32 normal samples. The corresponding clinical information of gastric adenocarcinoma patients was obtained from UCSC (https://xenabrowser.net/datapages/). Patients with incomplete clinical data and a follow-up time of <30 days were excluded. Gene expression profile data for GSE84437 were downloaded from the Gene Expression Omnibus (GEO) database (https://www.ncbi.nlm.nih.gov/geo/) and included 433 GC samples.

To convert the identification probes into gene symbols, the corresponding annotation files were used. The DEGs in GC were identified using the R software “limma” package with the criterion of |fold change| ≥1.5, and an adjusted P value < 0.05. Additionally, a list of 267 ferroptosis-related genes (FRGs) was downloaded from the FerrDb database (S1 Table; http://www.zhounan.org/ferrdb/index.html), which is an open-access resource that includes the regulators and markers of ferroptosis and ferroptosis-related diseases. Thus, the differentially expressed FRGs between GC tissues and normal gastric tissue were identified as candidate genes for further analysis based on the overlap between the DEGs in the GC tissue and normal gastric tissue obtained in the TCGA and GEO analyses, and the FRGs obtained from the FerrDb database. The TCGA dataset was used as the training cohort, and the GSE84437 dataset from the GEO database was used as the external validation cohort. Approval from the institutional ethics committee was not required for the current study, as the TCGA and GEO databases are publicly available.

### Risk score establishment

A univariate Cox regression analysis was used to select prognosis-related genes from the candidate genes. Subsequently, a LASSO Cox regression analysis was performed to select the optimal genes. Thus, a prognostic FRG signature that calculates individual risk scores was developed based on the nonzero coefficients in the LASSO regression model. The risk score was derived as follows: risk score = expmRNA1 × betamRNA1 + expmRNA2 × betamRNA2 + expmRNA3 × betamRNA3 + ⋯ + expmRNAn × betamRNAn. Here, “exp” represents the standardized expression of each identified mRNA and the “beta” was determined by LASSO Cox regression analysis. Patients with GC were dichotomized into high- and low-risk groups based on the median value of the risk score. The prognostic significance of the high- and low-risk score groups was then revealed using a Kaplan-Meier survival plot. Receiver operating characteristic (ROC) curves were used to evaluate the performance of the FRG signature. Consequently, we performed a principal component analysis (PCA) using the “prcomp” function of the “stats” R package and t-distributed stochastic neighbor embedding (t-SNE) with the “Rtsne” R package.

### External validation of prognostic FRG signature

The GSE84437 dataset was independently used for external validation. The same FRG signature was used to calculate the individual risk scores in the validation group. The performance of the FRG signature for predicting OS was validated using ROC curves and Kaplan-Meier analyses.

### Stratification analysis of the FRG signature

To explore the impact of the FRG signature on the clinicopathological features of GC, we evaluated the correlation of the risk score with seven clinicopathological factors (age, gender, tumor site, grade, tumor stage, T stage, and N stage). Tumor grade was defined according to the World Health Organization (WHO) classification scheme, and tumor stage was defined according to the TNM classification described in the seventh edition of the AJCC Cancer Staging Manual. In addition, a stratified analysis was used to assess whether the signature retained its predictive ability in various subgroups. These variables included age (<60 and >60 years), sex (female and male), tumor stage (I-II and III-IV), grade (G1-2, G3), T stage (T1-2 and T3-4), and tumor location (cardia and non-cardia).

### Immune infiltration analysis

To elucidate the correlation of the FRG signature in GC with immune infiltration levels, we calculated the infiltrating score of 16 immune cells, and 13 immune-related pathways were inferred from the GC gene expression profiles by a single-sample gene set enrichment analysis (ssGSEA) [11].

### Development of a prognostic nomogram

A Cox regression analysis was performed to evaluate whether the risk scores and clinical parameters (age, tumor site, grade, and tumor stage) were independent prognostic factors for OS. A nomogram incorporating the risk score and independent prognostic factors was developed using a multivariate Cox regression analysis to predict the probability of 3- and 5-year OS in patients with GC. The distinguishing ability of the nomogram was assessed using the area under the curve (AUC). Calibration plots were drawn to determine whether the predicted and observed probabilities of OS were in concordance.

### GO and KEGG enrichment analyses

The “limma” R package was used to identify the (DEGs) between the high-risk and low-risk groups with a criteria of |focal change| > 2 and P < 0.05. The GO enrichment and KEGG pathway analyses of the DEGs was conducted using the “clusterProfiler” package in R, with P < 0.05 considered statistically significant.

### Statistical analyses

The R Project for statistical computing (version 4.0.3) was used for the statistical analyses. Statistical significance was set at p < 0.05. The continuous variables of the two groups were analyzed using Student’s t test. Categorical variables were analyzed using the χ2 test or Fisher’s exact test. Immune infiltration analysis was investigated using the ‘gsva’ package in R. Univariate and multivariate Cox proportional hazards regression models were used to estimate hazard ratios and corresponding 95% confidence intervals (CI) for each potential prognostic variable. A Univariate survival analysis was performed for OS using the Kaplan–Meier method, and these factors were compared using the log-rank test (P < 0.05). The nomogram was created by the “Survival” and “RMS” packages of R 3.6.3 and could be used to provide a visual risk prediction.

## Results

### Clinicopathologic features of patients

**Fig 1:**
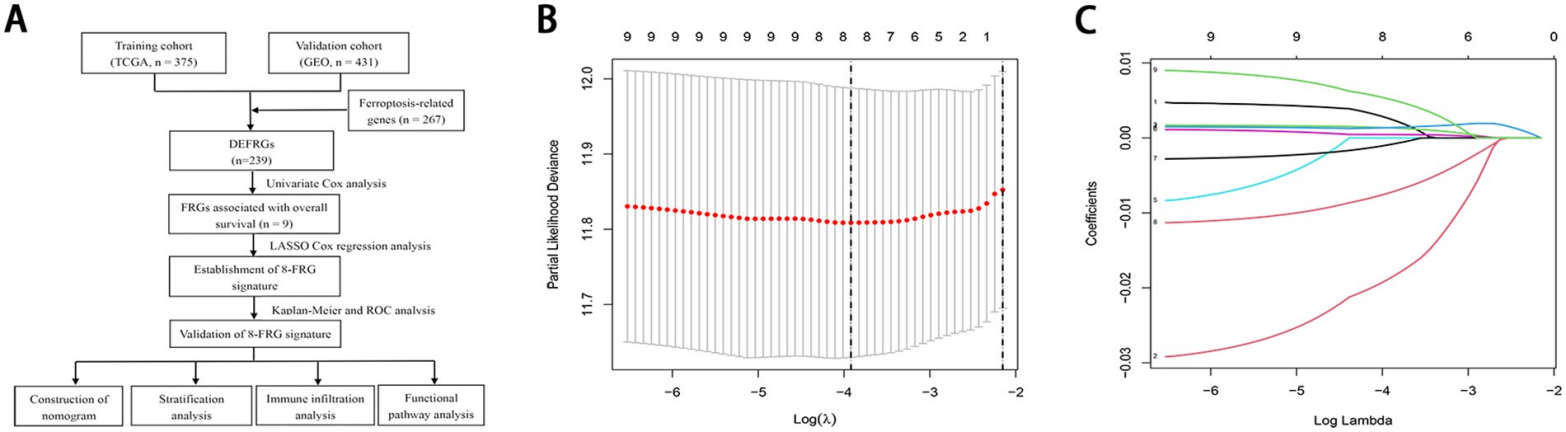
(A) Flow chart showing the development of the FRG signature and nomogram in GC patients (B) Ten-time cross-validation for tuning parameter selection in the LASSO Cox regression model (C) LASSO coefficient profiles of the 8 ferroptosis-related genes.

Fig 1A presents the study design. This study included 375 patients with GC from the TCGA database and 431 patients with GC from the GSE84437 dataset. In the training cohort, most patients (66.7%) were aged over 60 years, and the vast majority of GC patients were male, accounting for 64.0% of the cohort. Of the 375 GC patients, the patients’ pathologic stage was mainly G3 (62.7%); clinical stage was mainly concentrated in stage II (32.5%) and stage III (45.9%). Table 1 summarizes the baseline clinical features in the training and validation cohorts.

**Table 1:** Clinicopathologic characteristics of gastric cancer patients in TCGA and GEO cohorts.

Variables	TCGA cohort	GEO cohort
	(n = 375)	(n = 431)
	N (%)	N (%)
Age (M±SD, years)	65.22±10.51	60.02±11.58
Age		
≤60	125 (33.3)	194 (28.3)
> 60	250 (66.7)	237 (71.7)
Gender		
Female	135 (36.0)	137 (45.5)
Male	240 (64.0)	294 (54.5)
Tumor location		
Cardia	96 (25.6)	/
Non-cardia	279 (74.4)	/
Grade		
G1-2	140 (37.3)	/
G3	235 (62.7)	/
Stage		
I	47 (12.5)	/
II	122 (32.5)	/
III	172 (45.9)	/
IV	34 (9.1)	/

### Identification of differentially expressed FRGs in GC

After matching the gene expression data from the FRG list and the GSE84437 dataset, 229 FRGs were identified in the training cohort, with 61 differentially expressed FRGs. These differentially expressed FRGs served as candidate genes for further analyses.

### Development and validation of FRG signature

**Fig 2:**
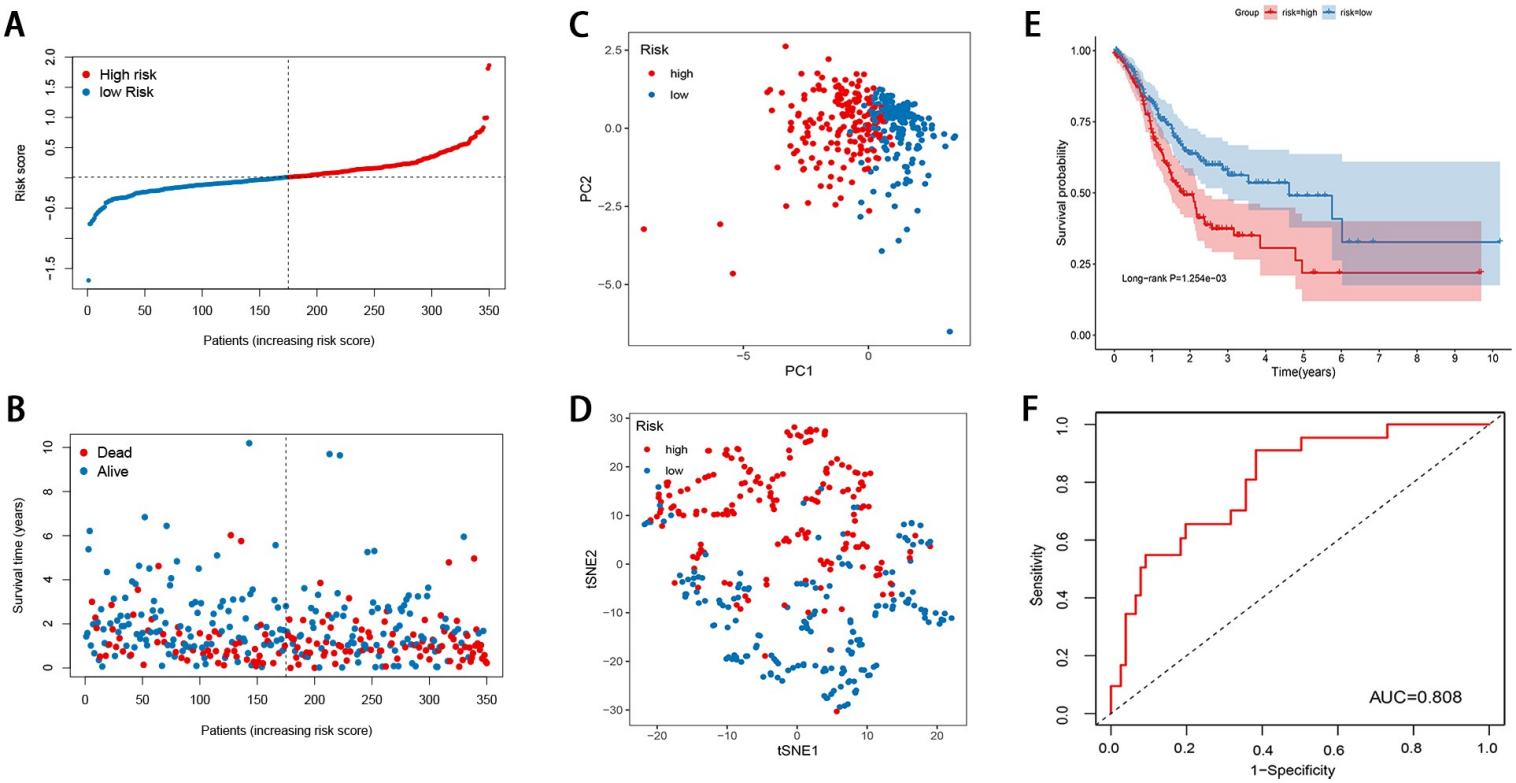
Construction of the ferroptosis-related gene signature in the training cohort (A) Risk score distribution (B) Overall survival time of GC patients (C) PCA plot (D) t-SNE analysis (E) Kaplan-Meier curves for the OS of patients in the high-risk group and low-risk group (F) ROC curve for the prediction of GC survival.

Nine prognostic FRGs related to OS were selected from the differentially expressed FRGs using the univariate Cox regression analysis. A LASSO Cox regression model was then built (Fig 1B and 1C), and eight FRGs were eventually identified, including GABARAPL1, ZFP36, DUSP1, TXNIP, NNMT, MYB, PSAT1, and CXCL2. The risk score was determined as follows: risk score = (˗0.0137 × expression of GABARAPL1) + (0.4796 × expression of ZFP36) + (0.2338 × expression of DUSP1) + (0.0462 × expression of TXNIP) + (0.0100 × expression of NNMT) + (˗0.0206 × expression of MYB) + (0.1205 × expression of PSAT1) + (0.3747 × expression of CXCL2). Based on the median value of the risk score, 350 GC cases were grouped into the high-risk (n = 175) and low-risk categories (n = 175). As illustrated in Fig 2A and 2B, the death sample survival time decreased with an increase in the risk score. Most patients who died belonged to the high-risk group. The PCA and t-SNE analyses indicated discernible dimensions in the high- and low-risk patients (Fig 2C and 2D). Further survival analysis was performed based on the risk score, and patients with a low-risk score had a better overall prognosis than those with a high-risk score (Fig 2E). To further assess the model performance, the time-dependent ROC was mapped. The AUC values were approximately 0.808 in the training cohort (Fig 2F).

### Validation of the FRG signature

**Fig 3:**
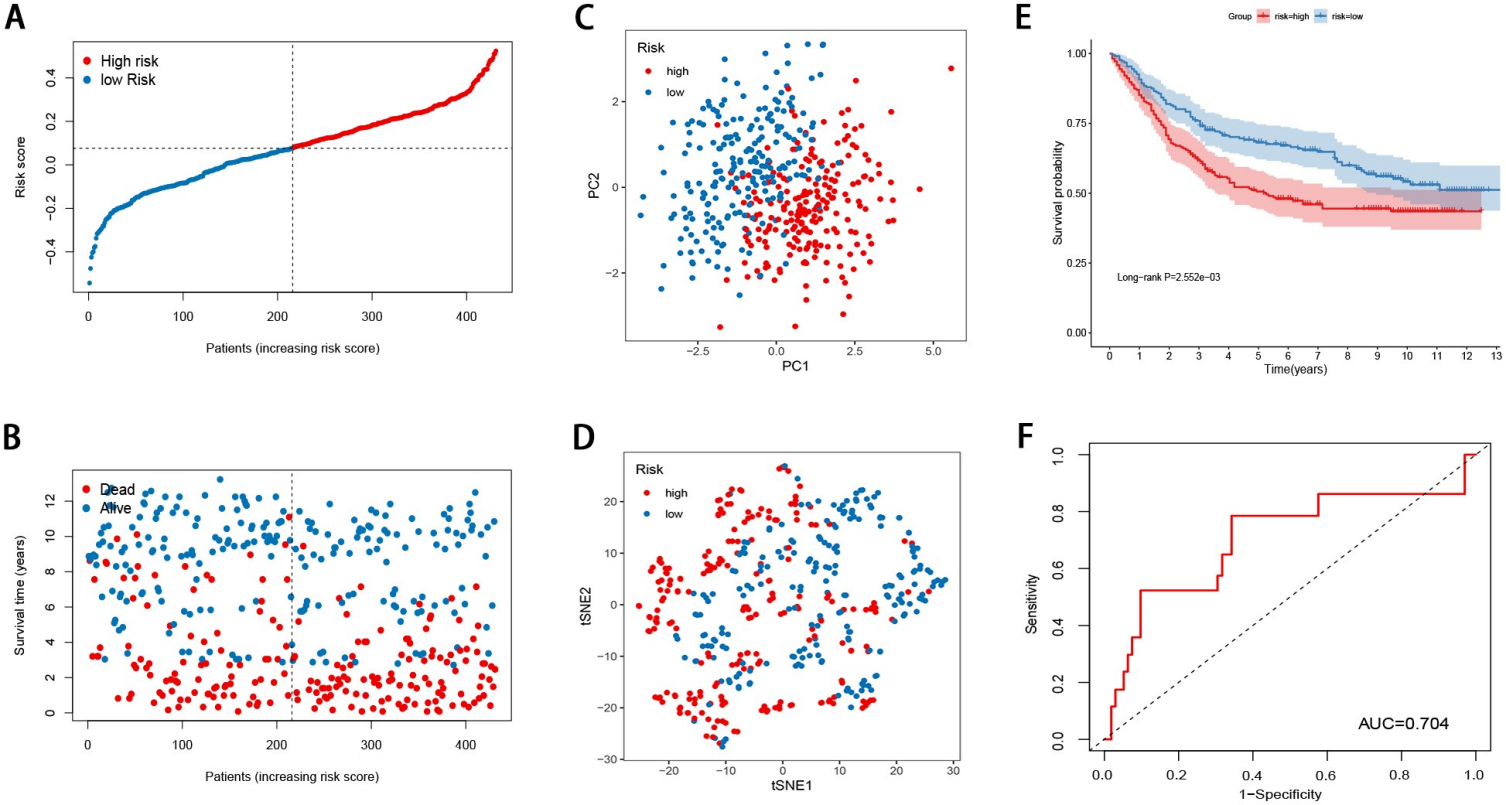
Validation of the ferroptosis-related gene signature in the validation cohort (A) The risk score distribution (B) Overall survival time of GC patients (C) PCA plot (D) t-SNE analysis (E) Kaplan-Meier curves for the OS of patients in the high-risk group and low-risk group (F) ROC curve for the prediction of GC survival.

The GSE84437 dataset was used to evaluate the performance of the FRG signature as an independent validation cohort (Fig 3A–3F). The risk score for each patient was calculated using the signature. The PCA and t-SNE analyses demonstrated that the patients in the different risk groups were distributed in two directions (Fig 3C and 3D). The Kaplan-Meier curves revealed that the FRG score was negatively correlated with a favorable prognosis (Fig 3E). The AUC of the FRG signature was 0.704 (Fig 3F). Taken together, the data of the independent validation cohort also presented a satisfactory performance of the FRG signature for survival prediction.

### Stratification analysis of the FRG signature

**Fig 4:**
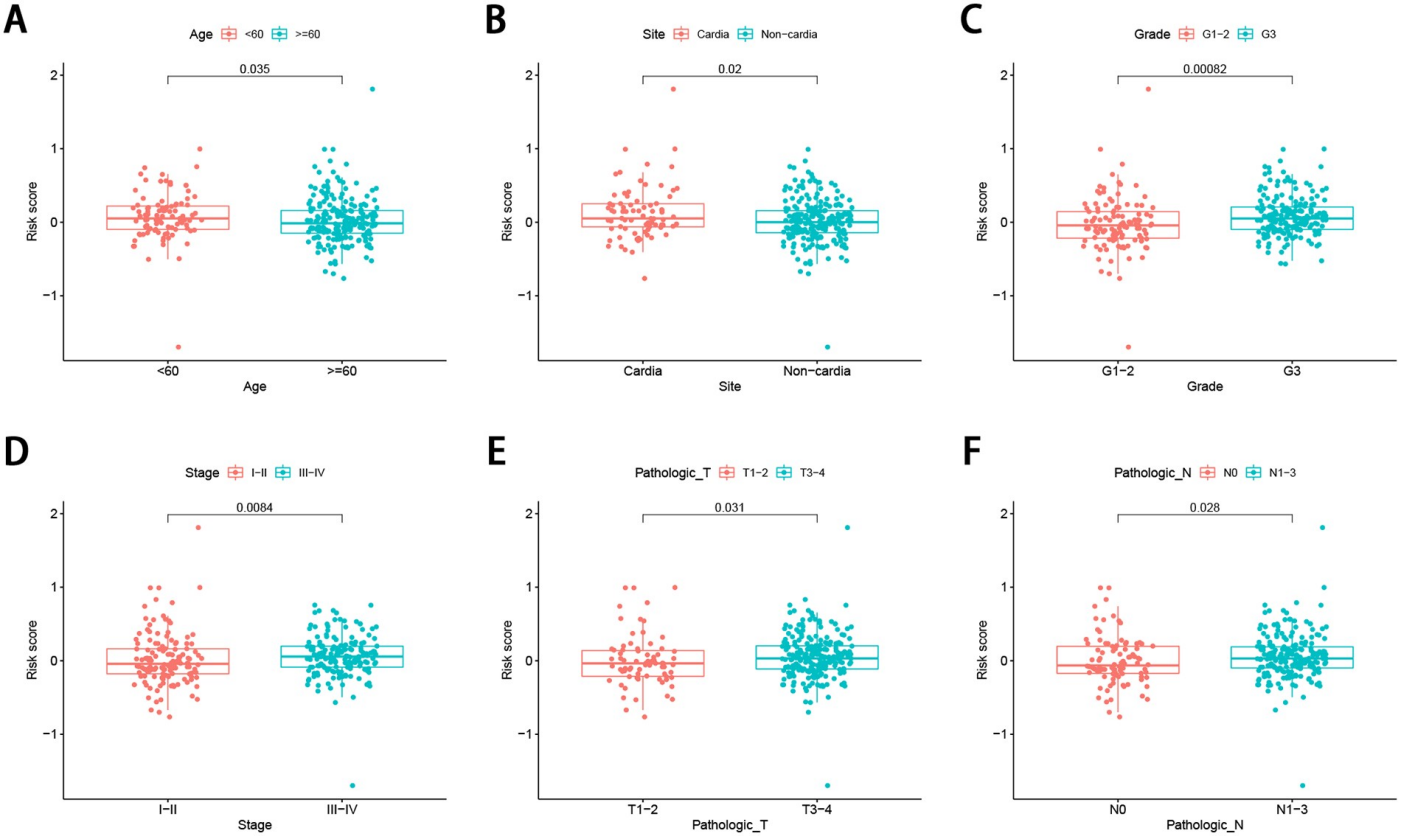
Association between the FRG signature and the clinicopathological characteristics (A) age (B) tumor site (C) grade (D) tumor stage (E) T stage (F) N stage.

**Fig 5:**
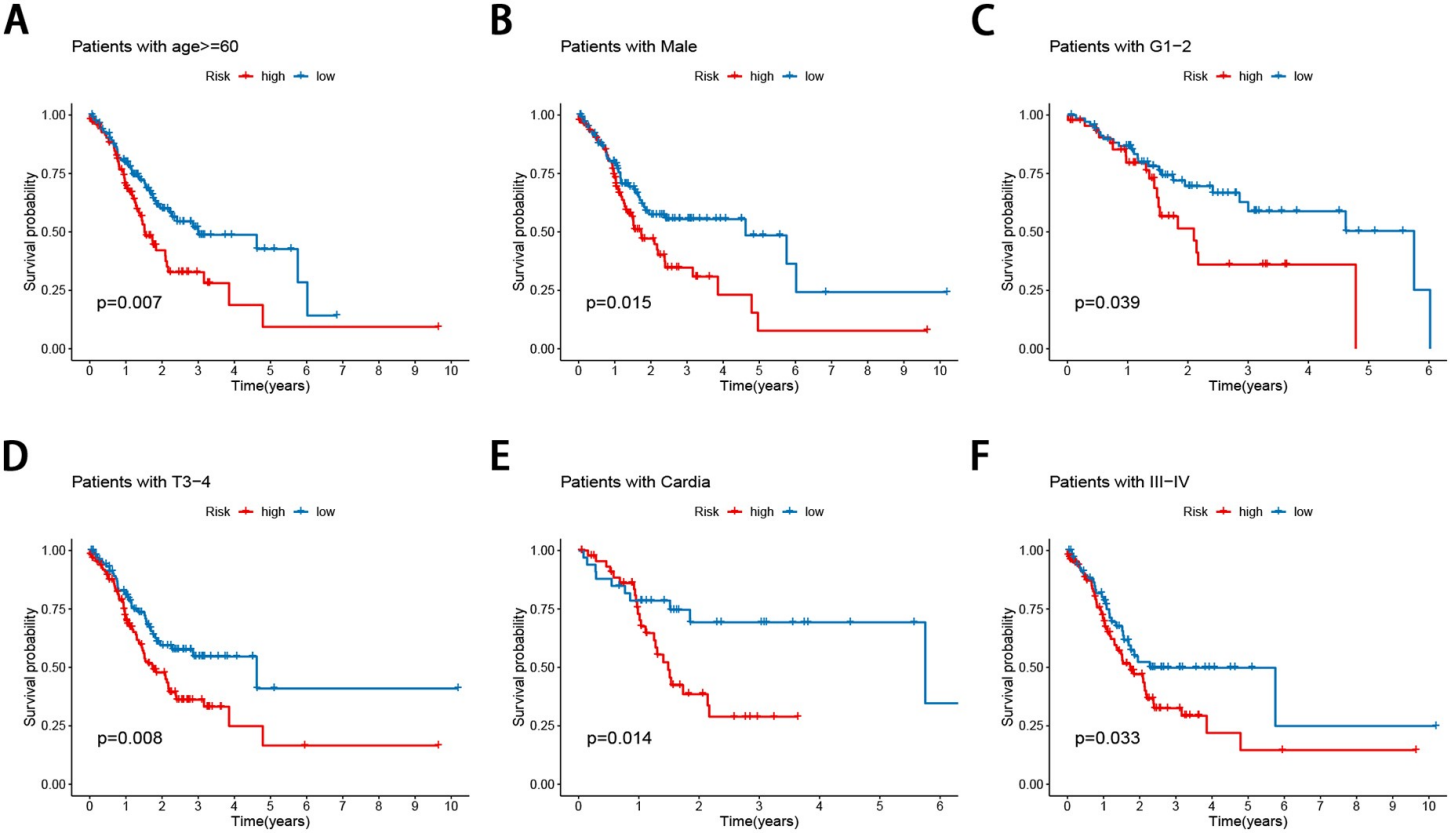
Stratification analyses by clinicopathological parameters. The survival difference between high- and low-risk group stratified by (A) age (B) gender (C) grade (D) T (E) tumor site (F) TNM stage.

The correlation of the risk score with the aforementioned seven clinicopathological factors was performed, and the results revealed that the FRG signature did not seem to be significantly related to sex, but was significantly related to age (Fig 4A), tumor site (Fig 4B), tumor grade (Fig 4C), tumor stage (Fig 4D), and T (Fig 4E), and N stage (Fig 4F) in GC, suggesting that the FRG signature may have an important impact on tumor progression. In addition, the prognostic value of the FRG signature for different clinicopathological parameters was further evaluated. Compared to patients with a lower risk, GC patients aged >60 years with a high risk had worse OS (Fig 5A), as did the male patient (Fig 5B), grade I and II (Fig 5C), stage T3-4 (Fig 5D), cardia site (Fig 5E), and stage III-IV (Fig 5F) subgroups.

### Immune infiltration analysis

**Fig 6:**
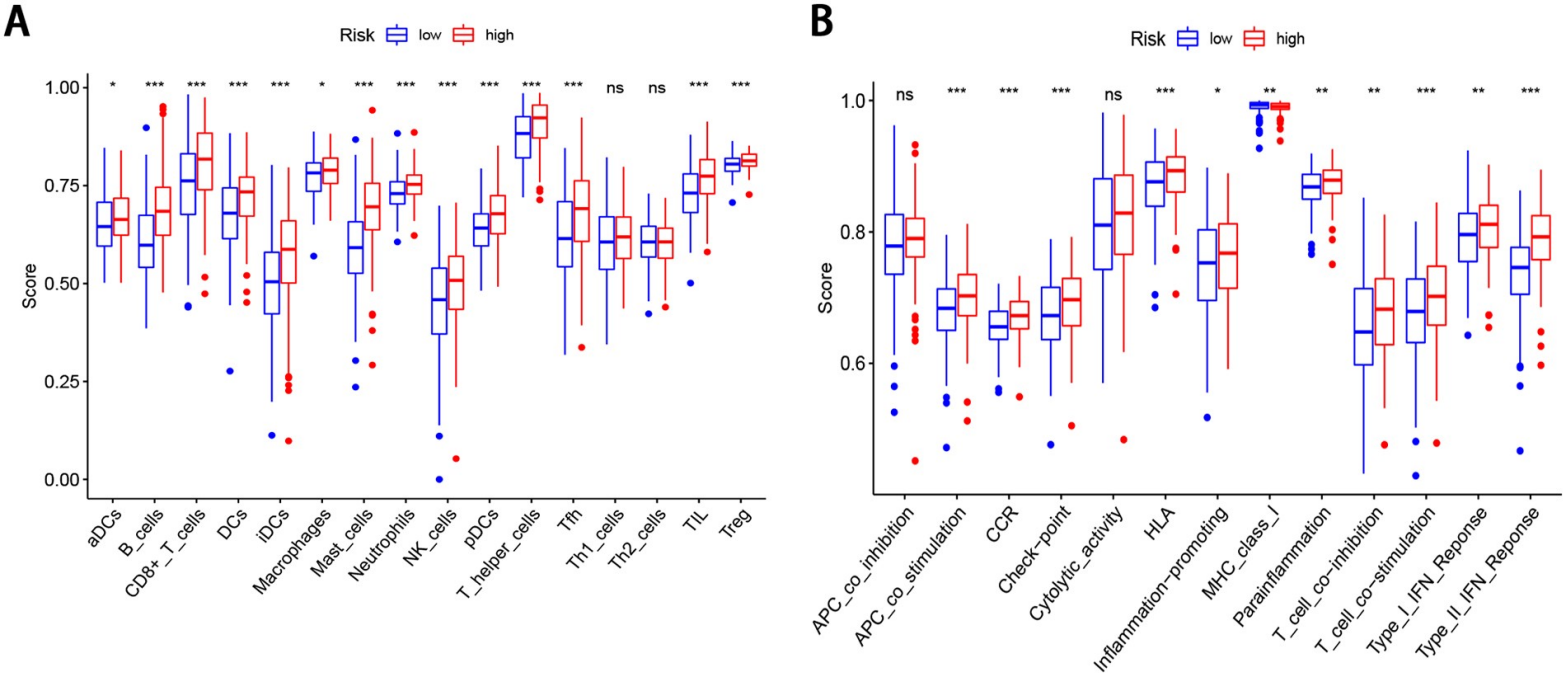
ssGSEA scores in the high- and low-risk patients in the TCGA cohort. The scores of 16 immune cells (A) and 13 immune-related functions (B) are displayed in boxplots.

To further explore the correlation of the FRG signature in GC with immune infiltration levels, we calculated the infiltrating score of 16 immune cells and 13 immune-related pathways using ssGSEA. The high-risk score was negatively associated with the scores of 12 immune cells, including aDCs, B cells, CD8+T cells, DCs, iDCs, macrophages, mast cells, neutrophils, pDCs, NK cells, T helper cells, Tfh, TIL, and Treg cells (P < 0.05, Fig 6A). In addition, 11 of the 13 immune-related pathways were significantly different in the high-and low-risk groups (P < 0.05, Fig 6B).

### Development of a prognostic nomogram

**Fig 7:**
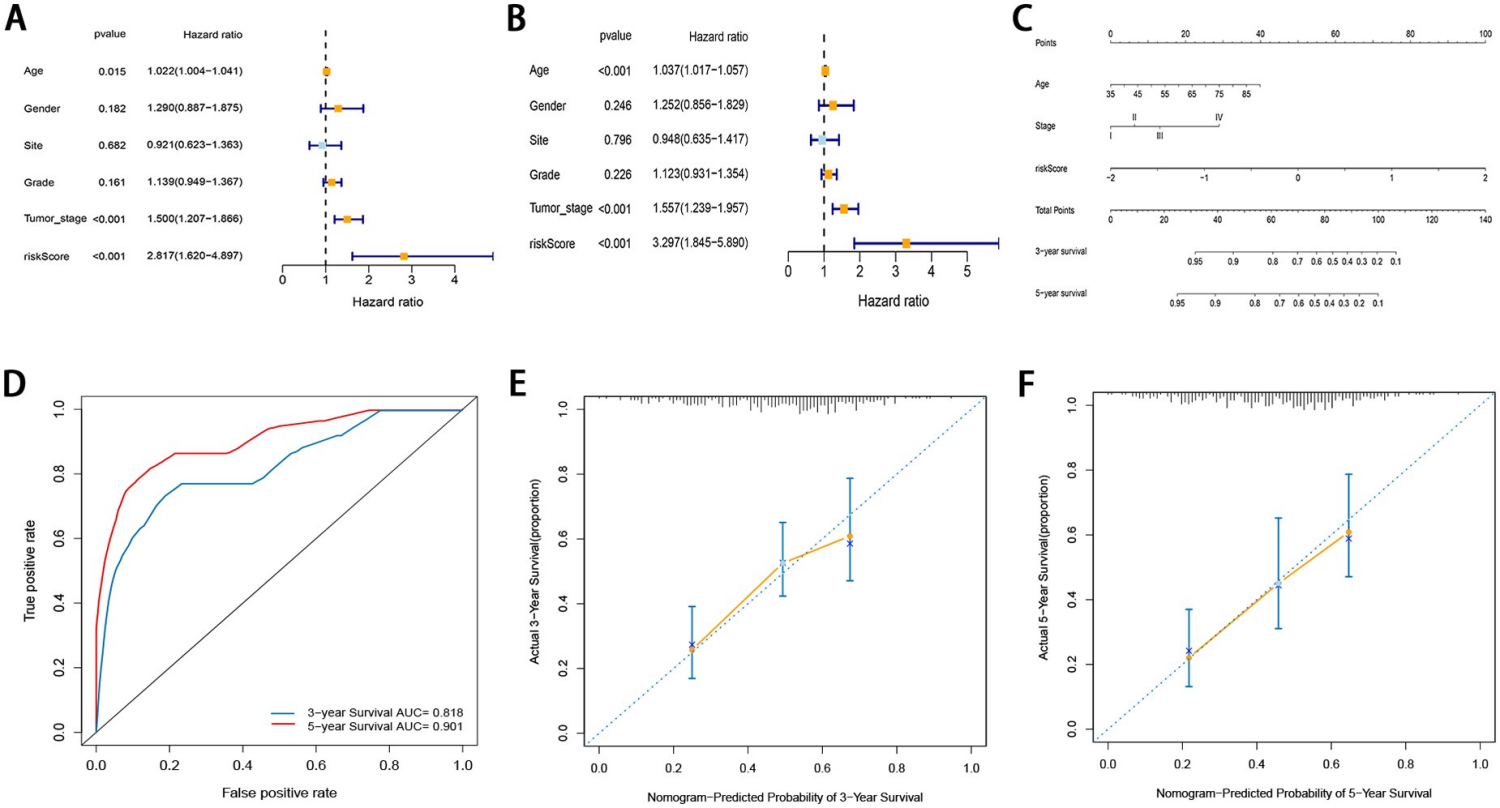
(A, B) Univariate and multivariate Cox regression analyses regarding OS in GC patients (C) Nomogram based on risk score, age, and tumor stage (D) Time-dependent ROCs for 3-year and 5-yearOS of the nomogram (E, F) Calibration curves of the nomogram prediction of 3-year and 5-year OS of patients with GC.

To determine whether the prognostic significance of this FRG signature depends on the clinicopathological parameters, univariate and multivariate Cox analyses were performed to analyze the following variables: risk score, age, tumor site, grade, and tumor stage (Fig 7A and 7B). The results of univariate and multivariate Cox analyses demonstrated that the risk score, age, and tumor stage were independent factors in the TCGA cohort. Even if the risk score was adjusted by other conventional clinicopathological factors, these three factors remained significant. A novel prognostic nomogram incorporating the three independent factors was developed based on the results of multivariate Cox regression analysis (Fig 7C). The possibility of the 3- and 5-year OS could be predicted by combining the scores associated with each variable and projecting the total score to the bottom and calculating the total score. With the help of the nomogram, the prognosis could be effectively predicted based on the individual characteristics of the patient. The nomogram was validated using time-dependent ROC curves and calibration curves. The 3- and 5-year AUC values of the nomogram for OS were 0.818 and 0.905, respectively (Fig 7D). The calibration curve also demonstrated high consistency between the actual proportion of 3-and 5‐year OS and the nomogram-predicted probability (Fig 7E and 7F).

### GO and KEGG pathway analysis

**Fig 8:**
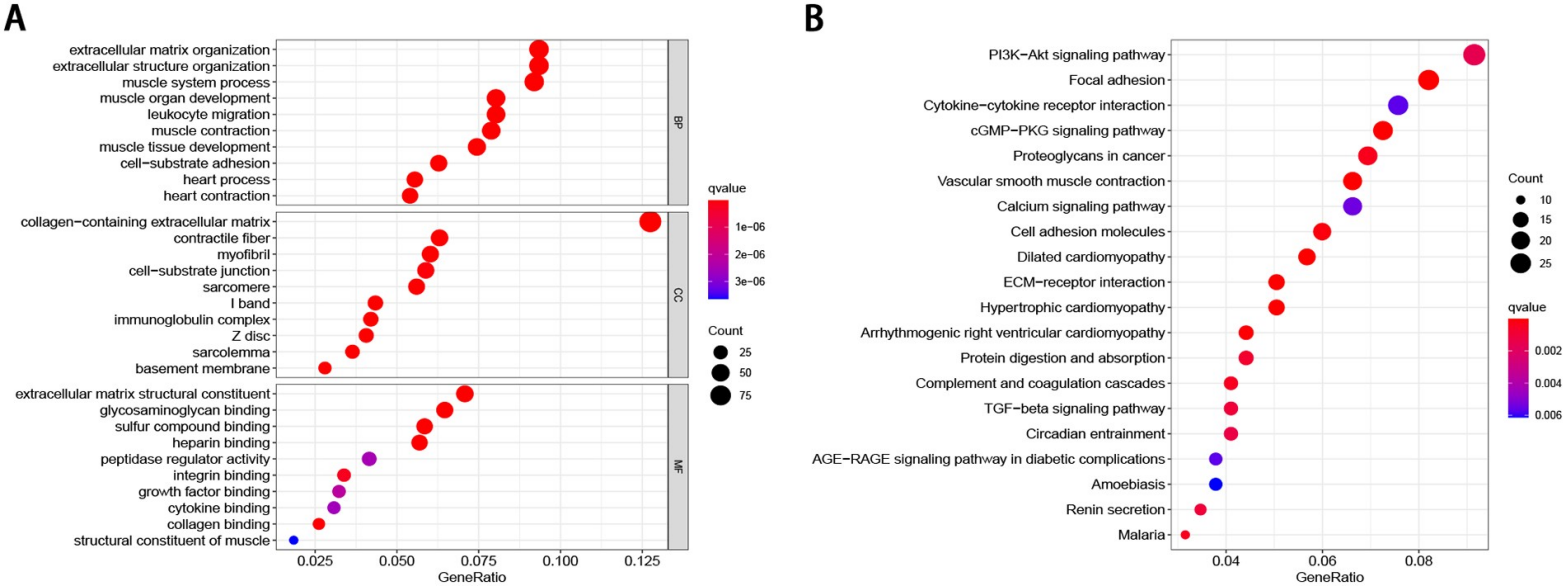
Functional enrichment analysis of the differentially expressed genes (A) Top 10 GO-enriched differentially expressed genes (B) Top 20 enriched KEGG pathways of the differentially expressed genes.

In order to identify the possible biological processes and signaling pathways in which the eight indicated FRGs may participate, we identified 804 differentially expressed FRGs between the low- and high-risk groups. The GO and KEGG analyses were performed based on these genes. In terms of the biological process, these genes were primarily enriched the extracellular matrix organization, extracellular structure organization, and muscle system processes. When considering cellular components, the most-enriched items were collagen-containing extracellular matrix, contractile fiber, and myofibril. With regard to molecular function, the extracellular matrix structural constituent, heparin binding, and glycosaminoglycan binding were enriched (Fig 8A). Meanwhile, the KEGG analysis revealed that the PI3K-Akt signaling pathway, focal adhesion, cytokine-cytokine receptor interaction, cGMP-PKG signaling pathway, MAPK signaling pathway, proteoglycans in cancer, vascular smooth muscle contraction, calcium signaling pathway, cell adhesion molecules, and dilated cardiomyopathy were the most frequently enriched pathways (Fig 8B).

## Discussion

GC in China seriously threatens the health and life of Chinese individuals, with a high incidence, low early diagnosis rate, and low survival rate [12]. Thus, it is important to screen specific cancer-related biomarkers for risk evaluation to predict the prognosis of patients with GC, and promote the development of effective therapies for GC. Cell death is crucial for the prevention of cancer; thus, the selective induction of cancer cell death is the most effective anticancer therapy. Ferroptosis reportedly plays a crucial role in tumorigenesis and cancer therapy. However, no FRG-associated biomarkers for predicting personalized survival have been developed. Given the potential influence of FRGs on the occurrence and development of GC, the identification of novel FRG-associated biomarkers may provide important prognostic information and therapeutic targets.

In this study, we developed a novel 8-FRG signature for predicting individual survival among patients with GC via an integrated analysis of the TCGA and GEO databases. Patients with a low-risk score had a better OS than those with a high-risk score. The predictability of the signature was confirmed by a tdROC curve, PCA, and t-SNE analyses, and an independent GEO dataset. Moreover, the signature showed a favorable predictive capacity in the stratification analysis. A high-risk score was associated with advanced clinicopathological features and an unfavorable prognosis. In addition, we identified differentially distributed immune cell immune-related pathways by ssGSEA and revealed that patients in the high- and low-risk groups had different levels of enrichment of immune cells and immune-related pathways. Functional enrichment analysis showed that DEGs were involved in many key biological functions related to tumor pathways, such as the PI3K-Akt signaling pathway, focal adhesion, MAPK signaling pathway, and in proteoglycans in cancer signaling pathways. Finally, the risk score, age, and tumor stage were identified as independent prognostic factors associated with OS in patients with GC, and a quantitative genomic-clinicopathologic nomogram incorporating these factors was developed to predict 3- and 5-year OS. The prognostic accuracy of the nomogram was confirmed using discrimination and calibration plots. The nomogram can provide personalized estimates of potential survival and can aid in individualized management decisions for GC.

Currently, the postoperative prognosis for GC is based on a comprehensive evaluation based on the AJCC staging system. This system is an excellent common language in the field of GC. However, even in patients with the same stage, the clinical behavior and prognosis of GC may vary. In addition, this staging system does not include prognostic factors such as age, tumor location, surgery type, or other tumor characteristics [13–15]. A nomogram is a visual representation of a mathematical model in which independent tumor-related factors can be used to estimate the specific endpoints of the model; it can simply and quickly predict clinical survival [16]. By integrating different independent prognostic factors, the nomogram can predict the possibility of an event outcome, such as the possibility of death or disease recurrence [17]. Several ferroptosis-related gene nomograms for predicting prognosis have been established for various types of cancer [18,19]. Zheng et al. [18] developed a 12-FRG signature to predict the OS of patients with lower-grade gliomas, which showed a favorable predictive capacity. Based on the signature and clinicopathological features, they established a nomogram for predicting 1-, 3-, and 5-year OS. Similarly, a prognostic nomogram integrating the FRG signature and patients’ clinicopathological information (age, T stage, and N stage) based on the expression levels of ferroptosis regulators was developed for pancreatic cancer [19]. To the best of our knowledge, FRG models or combined models built for predicting the outcomes of patients with GC have not been reported. Therefore, we established an 8-FRG quantitative signature for predicting the individual survival of GC patients and can aid in individualized therapy for GC.

A ferroptosis-related prognostic model for GC has recently been constructed [20]. Compared with this model, the model we built has some merits. The biggest merit of our study is that we constructed a quantitative genomic-clinicopathologic nomogram incorporating risk score and clinicopathological features that can provide personalized estimates of potential survival benefits and can aid individualized management decisions for GC. Moreover, the previous model was constructed using only 121 FRGs, while our model was constructed based on 267 recently reported FRGs. In both the training and validation groups, the AUC of the ROC curve in our model was significantly higher than that in the previous model, indicating that our model has a better predictive ability. The FRG signature proposed in this study was composed of eight FRGs (GABARAPL1, ZFP36, DUSP1, TXNIP, NNMT, MYB, PSAT1, and CXCL2). Similar to previous studies, our study revealed that GABARAPL1 is significantly involved in GC. GABARAPL1, which is a member of the GABARAP family, is highly evolutionarily conserved. Its coding gene was initially identified as an early estrogen-induced gene [21]. Gao et al. [22] revealed that GABARAPL1 is a potential positive regulator of ferroptosis via RNAi screening. However, they did not investigate the mechanism of its involvement in ferroptosis. Du et al. [23] reported that miR-143 inhibits autophagy by targeting GABARAPL1 in GC. In addition to its function in autophagy, GABARAPL1 has a significant influence on carcinogenesis; it is expressed at low levels in various types of cancers [24–26]. A study of a cohort of breast cancer biopsies demonstrated that lower GABARAPL1 expression in breast cancer was associated with a higher risk of recurrence [25]. Overexpression of GABARAPL1 can inhibit cell proliferation, colony formation, and invasion in breast cancer cells in-vitro [26]. ZFP36, also known as tristetraprolin, is an RNA-binding protein that induces a mesenchymal to epithelial phenotype by downregulating TWIST1 and SNAI1 [27]. Chen et al. [28] revealed that ZFP36 is downregulated in hepatocellular cancer tissues, which is consistent with our results. Further experiments have shown that ZFP36 can bind to PRC1 mRNA, thereby downregulating the expression of PRC1, inhibiting tumor growth, and promoting 5-Fu sensitivity. ZFP36 also reportedly protects against ferroptosis by regulating the autophagy-signaling pathway in hepatic stellate cells [29]. Accumulating evidence indicates that DUSP1 is involved in tumor cell proliferation, differentiation, transformation, cycle arrest, and apoptosis by regulating the MAPK signaling pathway [30,31]. It has also been reported that DUSP1 was closely associated with tumor cell resistance, including GC cells [32]. TXNIP is a 46 kDa multifunctional protein that participates in a variety of biological functions, including cell growth, differentiation, and energy metabolism [33]. TXNIP is considered a tumor suppressor in various tumors [34]. It has been proven that TXNIP deficiency promotes the development of GC via ROS signaling [35]. NNMT, which is a major metabolic regulator, is dysregulated and associated with a worse prognosis in various cancers, including GC [36,37]. Liang et al. [37] revealed that elevated NNMT in GC cells promotes epithelial-mesenchymal transition (EMT) by activating the expression of the transforming growth factor-β1. Another observational study revealed that exosomal NNMT from peritoneal lavage fluid could promote peritoneal metastasis in GC via TGF-β/smad2 signaling [38]. In addition, Zhang et al. found that NNMT was a ferroptosis-upregulating factor that affects ferroptosis and liver tumorigenesis in a GSH-dependent manner [39]. CXCL2 is a member of the CXC subfamily, which encodes secreted proteins related to immune regulation and mainly recruits neutrophils [40]. CXCL2 overexpression has been observed in several tumors, including GC. A study of 69 patients with GC demonstrated that the concentration of CXCL2 in the peripheral and tumor drainage blood was significantly higher than that in patients without recurrence [41]. Natsume et al. [42] indicated that the level of CXCL2 in the urine of GC patients with peritoneal metastasis was significantly higher than that in those without peritoneal metastasis, and the CXCL2-VEGFA axis plays a vital role in the peritoneal metastasis of GC. CXCL2 has also been found to affect tubule necrosis in oxalate nephropathy in a ferroptosis-dependent manner [43]. However, the effect of CXCL2 on tumor development in a ferroptosis-dependent manner has not yet been reported.

The GO analysis suggested that the ferroptosis-related gene signature is significantly associated with molecular function, which comprises extracellular matrix structural constituents, heparin binding, and glycosaminoglycan binding. The dysregulation of extracellular matrix deposition promotes BC aggressiveness by sustaining key growth, invasion, and survival pathways [44,45]. Glycosaminoglycans play an essential role in tumorigenesis and progression of BC [46–48], and represent biomarkers and targets for diagnosis, prognosis, and treatment. The KEGG analysis showed that the DEGs in the high-risk and low-risk patients identified in the present study were enriched in tumor-related pathways, including in the PI3K-Akt signaling pathway, focal adhesion, MAPK signaling pathway, and in proteoglycans in cases of cancer. The PI3K-AKT pathway is the most commonly activated pathway in human cancers. The oncogenic activation of the PI3K-AKT pathway in cancer cells reprograms cell metabolism by increasing the activity of nutrient transporters and metabolic enzymes, thereby supporting the anabolic needs of abnormally growing cells [49]. Zhang et al. [50] indicated that reduced m6A modification can predict malignant phenotypes and enhance Wnt/PI3K-Akt signaling in GC. Lin et al. [51] revealed that UFM1 inhibits the invasive activity of GC cells by attenuating the expression of PDK1 through PI3K/AKT signaling. Wu et al. [52] demonstrated that ORAI2 promotes the tumorigenicity and metastasis of GC through PI3K/Akt signal transduction and MAPK-dependent focal adhesion decomposition. Another study demonstrated that sinulariolide could inhibit the migration and invasion of GC cells by downregulating the EMT process and inhibiting the FAK, PI3K, AKT, and MAPK signaling pathways [53]. These findings of the functional enrichment analyses indicated that the eight FRGs may be responsible for GC progression by modulating various signaling pathways and biological processes.

Nevertheless, a few limitations of the present study need to be noted. First, the proposed prognostic model in the present study was established and validated using retrospective data from public databases. Future prospective studies are required to verify its clinical utility. In addition, the prognostic model used in this study was established using bioinformatics analysis. Therefore, further research is required to investigate the detailed mechanisms underlying the association between the identified FRGs and the individual survival of GC patients.

## Conclusions

We successfully developed a prognostic FRG signature and nomogram for OS in patients with GC. The novel FRG signature may serve as a reliable and reproducible tool for prognostic prediction in individual cases.

## Supporting information

S1 Table The ferroptosis-related genes. (DOCX)
