# Supplementary material for: Identification and validation of a novel ferroptosis-related gene model for predicting the prognosis of gastric cancer patients
Source: PLoS One. 2021 Jul 12;16(7):e0254368. doi: 10.1371/journal.pone.0254368 (PMC8274920; doi:10.1371/journal.pone.0254368)
Supplement: S1 Table — (DOCX) [file pone.0254368.s001.docx]

Table S1. The ferroptosis-related genes.

ACSL4

AKR1C1

AKR1C2

AKR1C3

ALOX15

ALOX5

ALOX12

ATP5MC3

CARS1

CBS

CD44

CHAC1

CISD1

CS

DPP4

FANCD2

GCLC

GCLM

GLS2

GPX4

GSS

HMGCR

HSPB1

CRYAB

LPCAT3

MT1G

NCOA4

PTGS2

RPL8

SAT1

SLC7A11

FDFT1

TFRC

TP53

EMC2

AIFM2

PHKG2

HSBP1

ACO1

FTH1

STEAP3

NFS1

ACSL3

ACACA

PEBP1

ZEB1

SQLE

FADS2

NFE2L2

KEAP1

NQO1

NOX1

ABCC1

SLC1A5

GOT1

G6PD

PGD

IREB2

HMOX1

ACSF2

CYBB

NOX3

NOX4

NOX5

DUOX1

DUOX2

VDAC2

PIK3CA

FLT3

SCP2

NRAS

KRAS

HRAS

TF

TFR2

SLC38A1

ATG5

ATG7

ALOX12B

ALOX15B

ALOXE3

G6PDX

ULK1

ATG3

ATG4D

BECN1

MAP1LC3A

GABARAPL2

GABARAPL1

ATG16L1

WIPI1

WIPI2

SNX4

ATG13

ULK2

EGFR

MAPK3

MAPK1

BID

CDKN2A

SOCS1

CDO1

MYB

MAPK8

MAPK9

MAPK14

LINC00472

PRKAA2

PRKAA1

ELAVL1

BAP1

MIR6852

ACVR1B

TGFBR1

EPAS1

HILPDA

HIF1A

IFNG

ANO6

LPIN1

HMGB1

TNFAIP3

TLR4

ATF3

ATM

YY1AP1

EGLN2

MIOX

TAZ

MTDH

IDH1

SIRT1

FBXW7

PANX1

DNAJB6

BACH1

LONP1

RB1

HSF1

SQSTM1

MUC1

SLC3A2

SLC40A1

FTMT

HSPA5

ATF4

HELLS

SCD

SRC

STAT3

PML

MTOR

TP63

CDKN1A

MIR137

ENPP2

FH

CISD2

MIR9-1

MIR9-2

MIR9-3

ISCU

OTUB1

LINC00336

BRD4

PRDX6

MIR17

SESN2

NF2

ARNTL

JUN

CA9

TMBIM4

PLIN2

MIR212

Fer1HCH

LAMP2

ZFP36

PROM2

CHMP5

CHMP6

CAV1

GCH1

DUSP1

NOS2

NCF2

MT3

UBC

ALB

TXNRD1

SRXN1

GPX2

BNIP3

OXSR1

SELENOS

ANGPTL7

DDIT4

LOC284561

ASNS

TSC22D3

DDIT3

JDP2

SLC1A4

PCK2

TXNIP

VLDLR

GPT2

PSAT1

LURAP1L

SLC7A5

HERPUD1

XBP1

ZNF419

KLHL24

TRIB3

ZFP69B

ATP6V1G2

VEGFA

GDF15

TUBE1

ARRDC3

CEBPG

SNORA16A

RGS4

BLOC1S5-TXNDC5

LOC390705

EIF2S1

KIM-1

IL6

CXCL2

RELA

HSD17B11

AGPAT3

SETD1B

FTL

MAFG

IL33

HAMP

DRD5

DRD4

MAP3K5

SLC2A1

SLC2A3

SLC2A6

SLC2A8

SLC2A12

GLUT13

SLC2A14

EIF2AK4

TFAP2C

SP1

HBA1

NNMT

PLIN4

HIC1

STMN1

RRM2

CAPG

HNF4A

NGB

YWHAE

GABPB1

AURKA

MIR4715

RIPK1

PRDX1

MIR30B
